# Patient involvement in quality improvement: a survey comparing naturalistic and reflective approaches

**DOI:** 10.1136/bmjoq-2022-001981

**Published:** 2023-05-16

**Authors:** Mattias Elg, Ida Gremyr

**Affiliations:** 1 Department of Management and Engineering, Linköping University, Linkoping, Östergötland, Sweden; 2 Technology Management and Economics, Chalmers University of Technology, Goteborg, Sweden

**Keywords:** Patient-centred care, Quality improvement methodologies, Evaluation methodology

## Abstract

**Background:**

This study investigates reflective and naturalistic approaches to patient involvement in quality improvement. The reflective approach, using, for example, interviews, provides insights into patient needs and demands to support an established improvement agenda. The naturalistic approach, for example, observations, is used to discover practical problems and opportunities that professionals are currently unaware of.

**Methods:**

We assessed the use of naturalistic and reflective approaches in quality improvement to see whether they differed in their impact on patient needs, financial improvements and improved patient flows. Four possible combinations were used as a starting point: restrictive (low reflective–low naturalistic), in situ (low reflective–high naturalistic), retrospective (high reflective–low naturalistic) and blended (high reflective–high naturalistic). Data were collected through an online cross-sectional survey using a web-based survey tool. The original sample was based on a list of 472 participants enrolled in courses on improvement science in three Swedish regions. The response rate was 34%. Descriptives and ANOVA (Analysis of Variance) in SPSS V.23 were used for the statistical analysis.

**Results:**

The sample consisted of 16 projects characterised as restrictive, 61 as retrospective and 63 as blended. No projects were characterised as in situ. There was a significant effect of patient involvement approaches on patient flows and patient needs at the p<0.05 level (patient flows, (F(2, 128)=5.198, p=0.007) and patient needs (F(2, 127)=13.228, p=0.000)). No significant effect was found for financial results.

**Conclusions:**

Moving beyond restrictive patient involvement is important to meet new patient needs and improve patient flows. This can be done either by increasing the use of a reflective approach or by increasing the use of both reflective and naturalistic approaches. A blended approach with high levels of both is likely to produce better results in addressing new patient needs and improving patient flows.

**WHAT IS ALREADY KNOWN ON THIS TOPIC**

- Patients are key actors in the creation, delivery and evaluation of healthcare services. They can be involved to various degrees, in different activities, and at different levels in healthcare quality improvement.

**WHAT THIS STUDY ADDS**

- This study adds knowledge by investigating the effects of naturalistic (eg, observation, video) and reflective approaches (eg, interviewing, focus groups) for patient involvement in quality improvement.

**HOW THIS STUDY MIGHT AFFECT RESEARCH, PRACTICE OR POLICY**

- A mix of working with high levels of use of both reflective and naturalistic methods enhances possibilities for quality improvements that result in new patient needs being met and patient flows being enhanced.

## Introduction

Patients are key actors in the creation, delivery and evaluation of healthcare services.^1 2^ Healthcare professionals increasingly involve patients in quality improvement (QI) in order to develop new services that better meet patients’ needs.^3 4^ Improvements in the quality of care can be achieved when the right method is used under the right circumstances.^5^ Patient-centric approaches to capturing patient experiences could be exemplified by allowing patients to describe their experience or by observing unfolding events—for example, through interviewing, directly observing patient behaviour or through a video-recorded message, where patients are able to provide insights into their journey from admission to discharge. Furthermore, patients can be involved to various degrees, in different activities and at different levels in healthcare QI.^6^ However, few studies have explored the actual impact or effects of patient involvement on QIs.^7–9^ Thus, little is known about the possible differences in impact of the various methods supporting patient involvement.

It has been shown that one of the most important phases of patient involvement is capturing the experiences^10^ of patients engaged in sharing their understandings, thoughts, problems and solutions. Thus, it is critical to place ‘the experience goals of patients and users at the centre of the design process and on the same footing as process and clinical goals’^11^ (p308). But, as many healthcare developers acknowledge, bringing the patient’s voice into the design and development process is difficult. Borrowing from the scholarly field of service research, this means overcoming difficulties in the identification of customers’ (here patients’) value-in-use, and shifting the focus from understanding the customer as a passive consumer towards the customer as an active participant in the value-creation process.^12 13^ In other words, and within a healthcare context, this means that ‘collaboration is promoted over passive patienthood’^14^ (p713) in the search for new, innovative and creative solutions. This also encompasses the emergence of new patient roles, ranging from participant to beneficiary of improvement work and from supplier to recipient of improvement outcomes.^15^

More active patient roles are at the heart of coproduction, which is ‘the interdependent work of users and professionals who are creating, designing, producing, delivering, assessing and evaluating the relationships and actions that contribute to the health of individuals and populations’^16^ (p 2). As coproduction is centred on interdependent work, changing patient roles naturally influence professionals’ roles, for example, moving from actions by professionals to create value for patients, to enabling work that supports patients in creating value and taking action themselves.^2^ In healthcare service coproduction, the cocreative relationships between patients and professionals are central. Such a relationship can occur at three levels: civil discourse, coplanning and coexecution^2^; indicating a range of approaches to the interaction ranging from being courteous and respectful, through truly trying to understand each other’s needs and values, and onwards to creating joint goals and sharing performance responsibility.

From a practical point of view, the move towards interactive, cocreative relationships has consequences for how patients’ experiences are captured, understood and used. There are also different ways to reach and elaborate on these experiences. One approach is to use methods that provide reflective patient accounts of events and experiences, for instance, through interviewing, using focus groups or doing social media analysis. These methods are backward-looking, that is, they aim to ‘discover, understand and satisfy the expressed needs’ of patients^13^ (p141) through reflective accounts. Another approach generates direct knowledge from the events and instances that patients are experiencing as their patient journey is unfolding. This naturalistic approach help to ‘discover, understand and satisfy the latent needs’ of patients^13^ (p141). This approach focuses on discovering problems that patients encounter in situ,^12^ for instance, through observation and diary (eg, a record of events, care episodes) methods. The reflective and naturalistic approaches, respectively, have different focuses, advantages and limitations for QIs,^17^ see table 1. Overall, a reflective approach is aligned with an already-established agenda for improvements, whereas a naturalistic approach is aligned with a more open approach, enabling patients to change an improvement agenda.^18^

**Table 1:** Comparison of reflective and naturalistic approaches to patient involvement

	Reflective	Naturalistic	References
**Focus**	Provide insights into patient demands and needs expressed through the meanings they attach to events and experiences	Open and focused on observable events in the context of the patient’s life as a way to discover practical problems and opportunities of which professionals are currently unaware.	13 30 31
**Role of patient**	Provide feedback about experiences in a supplier-like role.	Experiencing in situ with patients being active in conveying experiences.	32–35
**Role of professionals**	Listening and consulting; capturing experiences based on reflection-on-action.	Collaborative, enabling and co-designing together with patients, based on reflection-in-action.	11 25 27 36
**Examples of methods**	Interviews, focus groups, social media	Observations, video, diaries	12 13

Reflective and naturalistic approaches both have the potential to contribute to QI. However, while the reflective approach draws attention to accounts provided by patients when trying to describe and explain their experiences, naturalistic ways of working focus on what patients actually do as the event are unfolding. This is captured by the organisational learning scholar Argyris, who distinguishes between espoused theory, which represents people’s descriptions of how they intend to act in a given situation and the rationale behind these intended actions and theory-in-use, which reflects how people actually behave.^19^ What people say and what they do are two different things,^20^ mirroring the logics of the reflective and naturalistic methods.

Thus, hypothetically, the different types of method have different impacts on the outcomes of improvement work. The overall purpose of this paper is to understand the usefulness of naturalistic and reflective methods for patient involvement in QI. Specifically, the objectives of the current study were: (1) to measure the use of naturalistic and reflective methods in QI and (2) to assess whether these two types of method differ in their impact on the identification of patient needs,^10^ financial improvements^19^ and improved patient flows.^21^

## Methods

### Data collection

Data were collected through an online, cross-sectional survey using a web-based survey tool. The original sample was based on a list of 472 participants enrolled in courses on improvement science in three Swedish regions. Through snowball sampling, respondents in the original sample provided email addresses for 19 additional respondents. In all, 491 questionnaires were administered, and two reminders were sent by email, if required. In total, 155 respondents completed the entire questionnaire: a response rate of 32%. In addition, since several respondents (n=32) had retired, changed jobs or were on extended sick leave, the adjusted response rate was 34%.

### Analytical procedure

**Figure 1:**
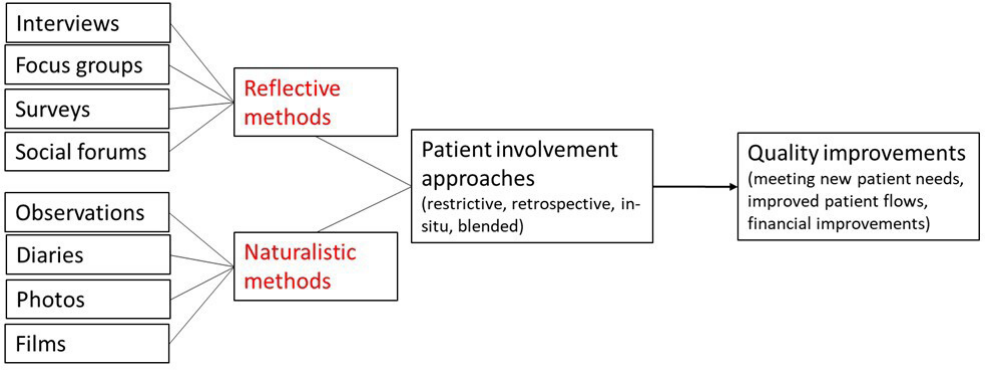
The conceptual logic of the study.

The analytical procedure of the work was conducted in the following order. First, we measured each method’s use through statistical calculations of average and SD. Second, as each respondent was asked to answer based on one, unique improvement project, we were able to identify each improvement project’s use of reflective and naturalistic approaches. This was measured on a dichotomous scale indicating low or high use. Thus, a particular project could potentially be characterised by low or high use of reflective approaches and low or high use of naturalistic approaches. Thus, there are four possible combinations, referred to as: restrictive (low reflective–low naturalistic), in-situ (low reflective–high naturalistic), retrospective (high reflective–low naturalistic) and blended (high reflective–high naturalistic). The labels for the combination with either low or high use of both reflective and naturalistic approaches are self-explanatory (overall low, ie, restrictive use or high use of both types of approaches, ie, a blended use). The in-situ label is chosen to emphasise the use of approaches that focus on collecting data during care, and retrospective the dominance of approaches to collect data after care. Third, we investigated the impact of the different combinations on three response variables, through Analysis of Variance (ANOVA) analysis. Fourth, we visualised the presence of the different combinations in different types of care. Figure 1 visualises the conceptual logic of the study and the analyses.

The use of reflective and naturalistic approaches was determined by adding up the different methods for each approach into an indicator score indicating low or high use. The survey question used was: ‘To what extent are the following methods used for capturing patient experiences?’ The survey items were measured on a 5-point Likert scale ranging from 1 to 5, with the category labels ‘not at all’, ‘a little’, ‘some’, ‘quite a lot’ and ‘a great deal’, with an option to mark ‘don’t know’.

A reflective approach is based on summing individual item ratings to an indicator score for the following items:

1. Interviews^13^
2. Survey^22^
3. Focus groups^23^
4. Social forums.^24^

A naturalistic approach is based on summing individual item ratings to an indicator score of the following items:

1. Observation^13^
2. Diary^12^
3. Photos^11^
4. Films^11^

In order to differentiate between high and low use of each approach (ie, the indicator score), a threshold was set at a minimum of 4 on the 5-point Likert scale for at least one of the included methods. For example, high usage of naturalistic methods was defined if a respondent ranked usage of observation as 4 and all others as 1. There were 15 missing values for this section in the survey which led to a total of 140 projects evaluated.

Each response variable (ie, meeting new patient needs, improved patient flows and financial improvements) was measured using a self-report, single-item measure. Meeting new patient needs was measured through the following survey question: ‘To what extent do you agree with the following statement: the new way of working has enabled us to meet patient needs that we did not try to meet earlier?’ This item was rated on a 5-point scale ranging from 1 (completely disagree) to 5 (fully agree).

Improved patient flows were measured through the survey question: ‘Based on your experience of this improvement project where patients/relatives have been involved—to what degree have patient flows been improved?’ Similarly, financial improvements were measured through the following survey question: ‘Based on your experience of this improvement project where patients/relatives have been involved—to what degree have finances been improved?”

A pilot questionnaire was evaluated by a focus group consisting of five healthcare professionals from different healthcare organisations with training and practical experience in patient involvement in QI. This contributed to clarifying the questions and instructions in the survey and ensured an understanding of the survey and its items among the focus group participants.

Descriptives were used to capture the various uses of reflective and naturalistic approaches to patient involvement. The first analysis revealed that there were three combinations: low-level use of both reflective and naturalistic approaches; low-level use of naturalistic and a high level of reflective and high levels of both. The effects of these three combinations in relation to the response variables were assessed using the ANOVA and post hoc tests. IBM SPSS Statistics 23 was used.

### Patient and public involvement

This study investigates QI projects led by healthcare professionals. A key issue in the research was to develop ways to assess the relationship between usage of various methods for patient involvement and their respective effects on three outcome variables (patient needs, financial improvements and improved patient flows). Research questions were developed based on the gap in the literature discussed earlier in the Introduction section. This required a design that covered the healthcare professional’s perspective through a questionnaire survey. Patients and public were not involved. Patients and public were not involved, which is further elaborated in the Limitations section.

## Results

About three out of four respondents were women (75.5%). Almost half of the respondents were nurses (45.8%), followed by 12.3% physicians. Other professions represented included physiotherapists (3.2%), occupational therapists (1.3%) and psychologists (1.3%). It is noteworthy that the category ‘other’ represents one-third of all respondents (32.9%).

Characteristics of the use of reflective and naturalistic approaches are provided in table 2. Among the reflective approaches, interviewing is the most frequently used method, followed by surveys. Focus groups are used to some extent, and social forums are only used in a few projects. Nine respondents scored social forums on the Likert scale intervals 4 and 5, implying that they used the method extensively.

**Table 2:** Descriptive statistics for reflective and naturalistic approaches

	Use of methods in patient involvement	N	Mean	SD
	Survey	137	3.61	1.66
Reflective approaches	Interviews	135	3.90	1.30
	Focus groups	124	2.35	1.58
	Social forums	113	1.40	0.95
	Observations	124	2.79	1.46
Naturalistic approaches	Diaries	114	1.56	1.08
	Movies	113	1.45	0.99
	Photos	115	1.37	0.93

**Figure 2:**
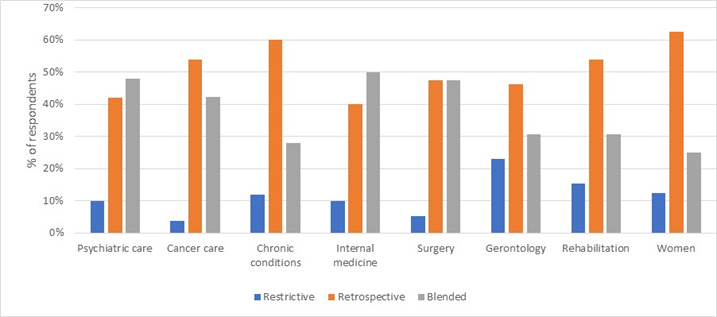
The use of various approaches to patient involvement in quality improvements in different care specialities.

Moving from individual methods to reflective and naturalistic approaches overall, 16 projects were characterised as restrictive, 61 as retrospective and 63 as blended. In our sample, no projects were characterised as in situ. Figure 2 displays the normalised percentages of restrictive, retrospective and blended approaches within various care specialities.

A one-way, between-subjects ANOVA was conducted to compare the effects of the four types of patient involvement approaches on financial results, patient flows and patient needs in the following method configurations (see table 3).

**Table 3:** Assessment of how different method configurations: restrictive (low reflective–low naturalistic); retrospective (high reflective–low naturalistic) and blended (high reflective–high naturalistic) influence the response variables

		Sum of squares	df	Mean square	F	Sig.
Financial improvements	Between groups	5078	2	2539	0.591	0.555
	Within groups	549 731	128	4295		
	Total	554 809	130			
Improved patient flows	Between groups	17 198	2	8599	5.198	0.007
	Within groups	211 733	128	1654		
	Total	228 931	130			
Meet new patient needs	Between groups	37 606	2	18 803	13.224	0.000
	Within groups	180 586	127	1422		
	Total	218 192	129			

Patient involvement methods had a significant effect on patient flows and patient needs at the p<0.05 level for all three configurations (patient flows (F(2, 128)=5.198, p=0.007) and patient needs (F(2, 127)=13.228, p=0.000)). No significant effect was found for financial results.

A post hoc comparison test (see online suppplemental appendix) for patient flows indicated that the mean score differed for restrictive versus retrospective and restrictive versus blended configurations. The post hoc comparison for patient needs indicated that all three types of approach are significantly different.

10.1136/bmjoq-2022-001981.supp1 Supplementary data

The use of reflective and naturalistic approaches varied in different types of care; see figure 1. The results from the profiles show that the most frequently represented type of care was mental health, followed by cancer care and chronic conditions. In mental health, the blended combination was most frequent (n=24). In cancer care, the most frequent combination was retrospective (n=14), although several respondents also reported the use of blended approach. In all types of care, restrictive combinations are low compared with the others. The retrospective and blended combinations vary between different types of care. For mental health, internal medicine and surgery, the blended approach is the most frequently used.

## Discussion

Previous research has highlighted the necessity of patient involvement at several levels of engagement, from direct care to organisational design and policymaking.^25^ Furthermore, patient involvement has also been argued to support professionals’ development as well as workplace developments. Examples of such support is to deepen the understanding of patients’ values and cultivating joint goals.^2^ The role of patient involvement in research has also been emphasised.^26^ At the same time, research has revealed uncertainty about how best to achieve patient involvement in practice.^3 12^ This includes, for instance, the design of engagement, the sampling of participants, leadership and lack of clarity of roles (see, eg, Bombard *et al*^27^ and Bergerum *et al*^6^ for recent reviews). As patient involvement implies putting things into practice,^28^ its supporting methods merit attention.

In general, there is a large body of research that provides evidence that patients both are willing and able to be involved in their care. However, when it comes to patient involvement in QI, empirical studies are scarce,^3^ especially when assessing the effects of various methods for capturing patients’ care experiences. This study set out to assess the use of reflective and naturalistic methods of patient involvement, as presented in table 1. Four means of patient involvement are outlined based on four combinations of the use of reflective and naturalistic methods: in situ (not represented in our data), blended, restrictive and retrospective. The findings show that the three patient involvement approaches represented in the data differ in their contributions to QI in terms of identifying new patient needs and improving process flows. However, the study also suggests that it is important to make a careful selection of which methods to use to support patient involvement adding to previous research on, for example, when to involve patients,^10^ or the role in which patients can be involved.^15^

In response to the purpose of understanding the usefulness of naturalistic and reflective methods for patient involvement, this study suggests that QI benefits from a high level of use of reflective and/or naturalistic methods. This finding is central because it underlines the necessity of inviting patients to participate. A restrictive approach is sometimes criticised as being exclusive and cosmetic and denoting tokenism.^25 29^ Our analysis confirms this, as a restrictive approach had limited impact on QIs.

The study also suggests that high levels of use of both reflective and naturalistic approaches in combination, that is, the blended approach, are more likely to generate better effects on meeting new patient needs and improving patient flows. There are several aspects that may be at play here, but one is the effectiveness of combining methods that naturalistically help to understand observable concrete practical events with reflective methods that enable patients to elaborate on the meanings of these events. When combining reflective and naturalistic methods, it is possible to understand what the problem is, and to understand from the patient’s perspective why it is a problem and how often it occurs. In other words, the combined use of different types of method enables patients to be involved in different roles.^15^ This makes it possible to actively involve them in a value-creation process^12 13^ based on a relationship between patients and professionals that become one of the improvement coexecutions.^2^

Finally, the use of the different types of approach to patient involvement varied depending on the type of care. Due to the small size of our sample, it was not possible to estimate the effects within each type of care, but it might be that certain approaches to patient involvement are more feasible in particular types of care. It would be of interest for future research to further investigate the applicability and impact of different approaches to patient involvement in different types of care specialities, and for different patient groups.

In conclusion, moving beyond restrictive patient involvement appears to be what matters in order to meet new patient needs and increase patient flows. This can be done either by increasing the use of reflective methods (ie, applying a retrospective approach) *or* by increasing the use of both reflective and naturalistic methods (ie, applying a blended approach).

### Limitations

Focusing on patient involvement, a limitation of this study is that the survey respondents did not include any patients. Thus, an area of future research is to include patients either in evaluating the type of approaches to patient involvement, or by evaluating the perceived outcome through patient-reported outcome and patient-reported experience measures. Moreover, a potential bias could have been present in the respondents’ interpretation of diaries in terms of extent of reflective entries. For cocreation to be realised, there is a need for new roles of both patients and professionals^2^ and, thus, both groups need to be open to involvement. To capture the professionals’ perspectives on involvement, this study employs subjective measures, such as self-reported assessments of practical engagement in QI, which is a topic of much discussion. We ensured the validity of the survey by employing the following strategies. First, all the questions were based on instruments with established validity; second, question wording was carefully chosen to reflect commonly understood terminology; third, and most importantly, focus groups were conducted before administering the survey in order to evaluate how professionals interpreted the survey questions.

Although a low response rate—in our study 34%—not necessarily lead to biased results, our results cannot broadly be generalised. In addition, as the survey is limited in the number of respondents and to a specific national context, a broader survey would be a useful next step in future research. However, in conclusion, the results show that a blended way of working with high levels of use of both reflective and naturalistic methods enhances possibilities for QIs that result in new patient needs being met and patient flows being enhanced.

## Data Availability

Data are available upon reasonable request.
