# Supplementary material for: Patient involvement in quality improvement: a survey comparing naturalistic and reflective approaches
Source: BMJ Open Qual. 2023 May 16;12(2):e001981. doi: 10.1136/bmjoq-2022-001981 (PMC10193082; doi:10.1136/bmjoq-2022-001981)
Supplement: Supplementary data [file bmjoq-2022-001981supp001.pdf]

Table Appendix: Post hoc tests

Dependent Variable			Mean Difference	Std. Error	Sig.	95% Confidence Interval Lower Bound	95% Confidence Interval Upper Bound
Financial improvements	LL	HL	-0.445	0.634	0.484	-1.70	0.81
		HH	-0.668	0.636	0.295	-1.93	0.59
	HL	LL	0.445	0.634	0.484	-0.81	1.70
		HH	-0.224	0.382	0.559	-0.98	0.53
	HH	LL	0.668	0.636	0.295	-0.59	1.93
		HL	0.224	0.382	0.559	-0.53	0.98
Improved patient flows	LL	HL	-LL0*	0.395	0.012	-1.78	-0.22
		HH	-1.267*	0.393	0.002	-2.05	-0.49
	HL	LL	LL0*	0.395	0.012	0.22	1.78
		HH	-0.267	0.237	0.262	-0.74	0.20
	HH	LL	1.267*	0.393	0.002	0.49	2.05
		HL	0.267	0.237	0.262	-0.20	0.74
Meeting new patient needs	LL	HL	-1.250*	0.338	0.000	-1.92	-0.58
		HH	-1.724*	0.337	0.000	-2.39	-1.06
	HL	LL	1.250*	0.338	0.000	0.58	1.92
		HH	-0.474*	0.223	0.036	-0.92	-0.03
	HH	LL	1.724*	0.337	0.000	1.06	2.39
		HL	0.474*	0.223	0.036	0.03	0.92

*. The mean difference is significant at the 0.05 level.
